# Osteomyelitis of the Knee Following Intra-Articular Injections: A Case Series

**DOI:** 10.31729/jnma.4858

**Published:** 2020-03-31

**Authors:** Anand Sobhraj Devnani, Raymond Dieu Kiat Yeak, Che Hamzah Fahrudin

**Affiliations:** 1 Department of Orthopaedic Surgery, Faculty of Medicine and Health Sciences, Universiti Putra Malaysia, 43400 Serdang, Selangor, Malaysia

**Keywords:** *intra-articular injection*, *knee*, *osteoarthritis*, *osteomyelitis*, *septic arthritis*

## Abstract

Septic arthritis of the knee is rare in adults. This leads to difficulty in making early diagnosis that invariably leads to delayed treatment with consequent destruction of the joint. The delay in diagnosis is largely attributed to absence of clinical signs of flagrant infection. Reported are three adult patients who presented with painful swollen knee and inability to walk few weeks after intraarticular injection for osteoarthritis. This paper discusses the cases in which the difficulties in the early diagnosis of septic arthritis of knee in adults led to the development of osteomyelitis.

## INTRODUCTION

Septic arthritis of the knee in adults is rare.^1,2^ This rarity leads to difficulty in making early and accurate diagnosis that invariably leads to delayed treatment with consequent destruction of the joint.^2^ The delay in diagnosis is largely attributed to absence of clinical signs of fla grant infection unlike that in children. Reported are three adult patients who presented with painful swollen knee and inability to walk few weeks after intra-articular injection for osteoarthritis by their family doctors.^3,4^ This paper discusses the difficulties in the early and accurate diagnosis of septic arthritis of knee in adults.

## CASE 1:

**Figure 1-A., Figure 1-B., Figure 1-C.**
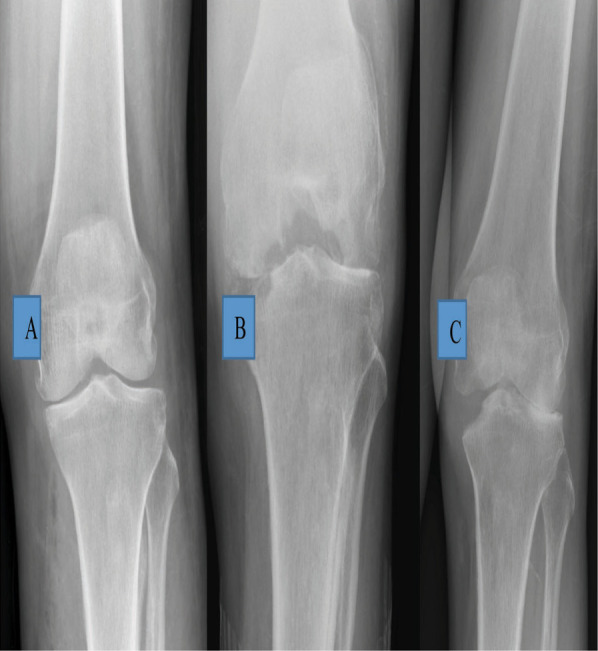
Radiograph (taken elsewhere) - AP view of the left knee just before the last intra-articular injection was given, showing that the medial joint space is narrowed, but the femoral and tibial condyles are not destroyed, Radiographs AP and lateral views of the same knee taken a month later, on admission, show marked destruction of femoral and tibial condyles. Such rapid destruction of bone is commonly seen due to pyogenic infection. Posterior subluxation of tibia seen in Lateral view, AP radiographs of the both knees 9 months post-arthrotomy and antibiotic therapy, shows some reformation of the medial femoral and tibial condyles.

A housewife aged 58 years, controlled diabetic, with osteoarthritis of both knees presented with painful left knee for past three months and inability to walk for one week. She denied any injury or constitutional symptoms. For past 3 years her family doctor had treated her for osteoarthritis and had injected the left knee several times (Figure 1-A).

The last injection was a month prior to her presentation. She came to orthopaedic clinic in a wheelchair, did not look unwell and was afebrile. The knee was tender over medial aspect and was kept in flexed position. The movements of the knee were painfully restricted from 300 to 900 of flexion. She refused to walk because of pain. The right leg was normal. Radiographs showed erosion of the medial tibial and femoral condyle of the femur with reduced joint space. (Figure 1-B). After blood investigations (Table 1) and blood-stained aspiration of the knee joint,provisional diagnosis of septic arthritis of the left knee was made and intravenous cloxacillin and gentamicin were started.

**Table 1:** Patient characteristics and results of investigations.

Parameter	Case 1	Case 2	Case 3
Age	58 years	48 years	69 years
Gender	female	female	male
How Arrived	In wheelchair	In wheelchair	crutches walking
Duration of symptoms	4 weeks	4 months	3 weeks
Blood Hb (mg/dL)	11.5	6.7	11.7
WBC (x109/dL)	13.5	6.7	9.8
ESR (mm /h)	120	80	50
CRP (mg/L)	275	28.52	13.01
Creatinine (µ mol/L)	74	70	75
Knee joint aspirate	Blood stained	Blood stained	Blood stained
Aspirate culture	no growth	no growth	no growth
Blood culture	“	“	“
Pus culture	“	“	“
Tissue culture	Staph. aureus	“	“
Histopathology	Acute infection	Acute infection	Chronic infection
Radiology	Rapid bone destruction & formation	Rapid bone destruction & formation	Rapid bone destruction

Next day, arthrotomy of the left knee was done. About 20cc of haemo-purulent fluid was drained and joint was thoroughly washed with saline. The synovium was inflamed. The blood and pus culture showed no growth.

The synovial tissue grew staphylococcus aureus which was sensitive to cloxacillin and gentamicin. The histopathological examination of synovial tissue showed acute on chronic inflammation with no granuloma or features of malignancy. She was given antibiotics for total of 6 weeks. She had started walking with a frame after 2 months. Ten months post-op, she was pain free, wore a knee brace and was walking with a frame. The radiographs (Figure 1-C) showed some reformation of femoral and tibial condyles.

## CASE 2

A woman aged 48 years, with no known medical illness, presented with painful swollen left knee for past four months. She sprained her left knee while getting up from kneeling position four months ago. As the pain had worsened, she consulted her family doctor who aspirated and injected the left knee. This did not relieve the pain. She came to the clinic in a wheelchair, was not unwell and was afebrile.The knee was warm and tender with painfully restricted range of flexion from 15 to 90. The radiographs and magnetic resonance imaging (done previously) showed osteomyelitis changes involving the patella, proximal tibial and the distal femur with reduced joint space. (Figure 2-A & B).

**Figure 2-A:**
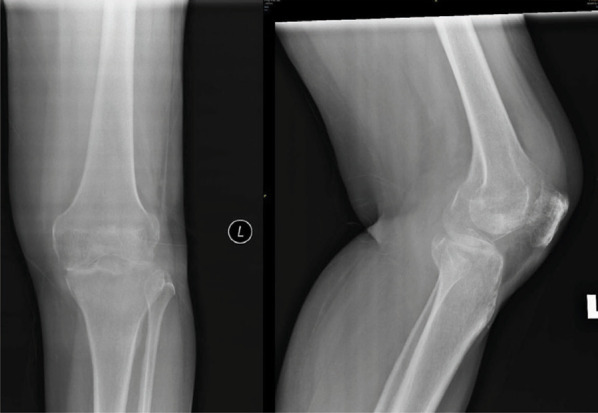
Radiographs AP and lateral views of the left knee, on admission show erosion of the medial tibial and femoral condyle with reduced joint space.

**Figure 2-B:**
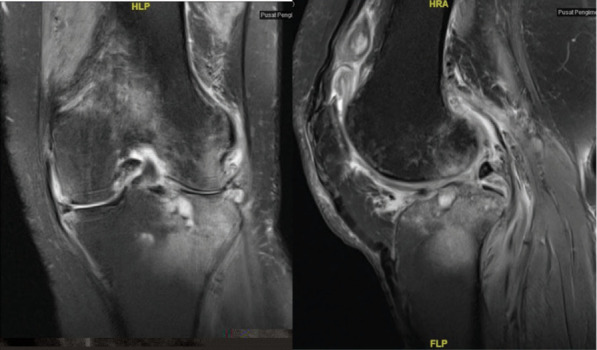
MRI (coronal and sagital) showing osteomyelitis of the medial tibial and femoral condyles.

**Figure 2-C:**
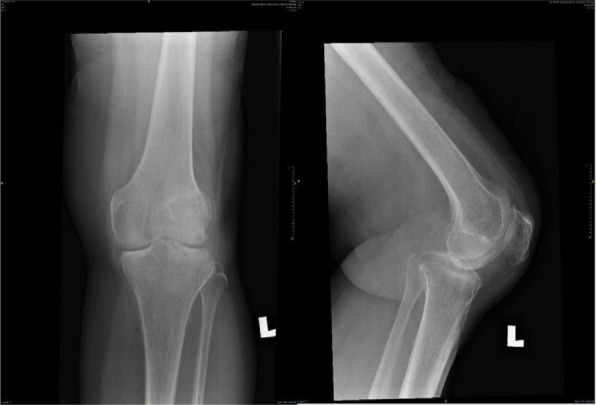
Radiographs AP and lateral views of the left knee, 7 months post-arthrotomy and antibiotic therapy, shows some reformation of the medial femoral and tibial condyles.

After blood investigations (Table 1) and aspiration of the knee, diagnosis of septic arthritis was made. Intravenous cloxacillin and gentamicin were started. On arthrotomy of the knee no haemo-purulent fluid was found but the synovium was inflamed. The blood and tissue cultures were sterile. The histopathological examination showed granulation tissue with chronic inflammatory process. No evidence of chronic granuloma or malignancy was seen. Antibiotics were continued for six weeks. At two weeks follow-up, patient was walking with a stick. At 4 months, she was walking using a stick, the left knee was not painful, and she could actively flex from 0 to 120. The radiographs done 7 months post-op (Figure 2-C) showed some reformation of femoral and tibial condyles.

## CASE 3:

A man aged 69, retired salesman of food products, presented with complaints of inability to stand on his left knee for 3 weeks. Three weeks ago, while standing up from sitting position, he felt his left knee could not support his weight. He prevented himself from falling by holding on to the rail. An hour later, he could get up and drove his car back home. Two weeks later he consulted his family doctor who advised for a radiograph of the left knee. About a year ago, he was given intra-articular injection to the left knee by his doctor. He presented to the orthopaedic clinic walking with crutches along with the recent radiograph. He was afebrile and well. The left knee was swollen but did not look inflamed and was not tender. He could actively flex the knee from 0 to 120. He had 100 of hyperextension and 200 each of valgus and varus laxity. Varus-valgus stressing of the left knee did not cause any pain. No distal neurovascular deficit was detected. Radiograph of the knee on admission showed (Figure 3-A) remarkably greater destruction of both femoral and tibial condyle as compared with radiograph done 2 weeks earlier. MRI (Figure 3-B) showed marked destruction of the tibial plateau and of the femoral articular cartilage.

**Figure 3-A:**
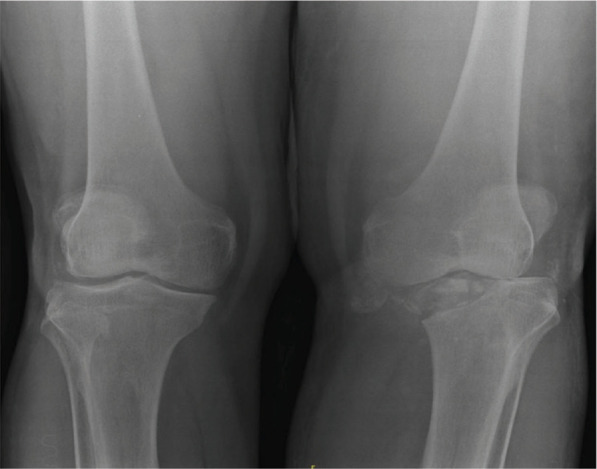
Radiographs AP view of both knees, on admission showing marked destruction of medial tibial plateau of the left knee. There is lateral subluxation of the tibia.

**Figure 3-B:**
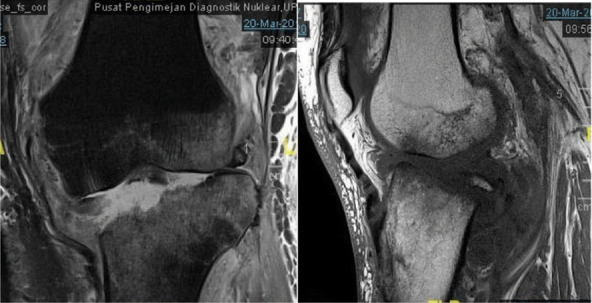
MRI scan of the left knee showing marked destruction of the tibial plateau in the coronal view. The sagittal view shows femoral condyle is also involved.Involvement of both distal and proximal surfaces of the joint strongly suggest infective pathology.

**Figure 3-C:**
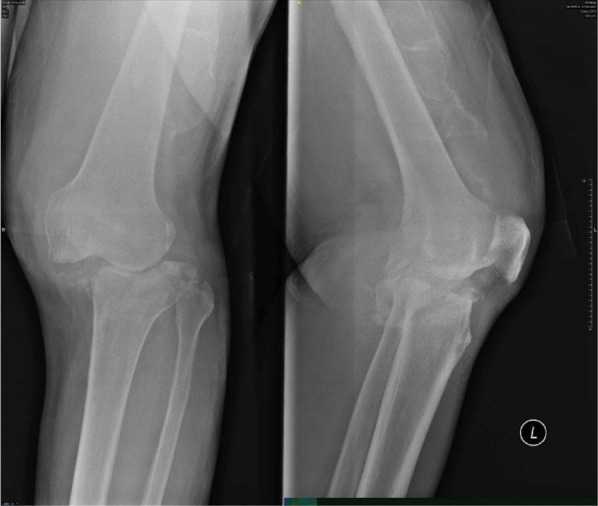
Radiographs AP and lateral views of the left knee, 2 months post-arthroscopic washout and antibiotuc therapy, shows some reformation of the medial femoral and tibial condyles.

After blood investigations (Table 1) diagnosis of left knee septic arthritis was made. Intravenous cloxacillin was started. On arthroscopy, both the menisci along with ACL and PCL ligaments were destroyed. The synovium was inflamed. The tibial plateau and both femoral condyles were damaged. Twenty millimeters of haemo-serous fluid was drained, and the joint was washed with saline. The blood, tissue and bone culture showed no growth. The synovial histopathology showed granulation tissue with chronic inflammatory process with no evidence of chronic granuloma or malignancy was seen. The patient completed 2 weeks of intravenous cloxacillin. At two months follow-up, patient was walking with a stick. The radiographs done 2 months post-op (Figure 3-C) showed very little bone reformation.

## DISCUSSION

Isolation of the causative microorganism from the culture of the aspirated joint fluid is considered benchmark index for the diagnosis of septic arthritis. However, pus or fluid culture is positive in less than half the patients possibly due to previous antibiotic therapy or the causative micro-organism is of low virulence whose culture growth may require longer incubation period.2 Both women, cases 1 and 2, had the last intraarticular injection few weeks prior to presentation. Case 3 gave history of injections nearly a year ago. The pus culture was sterile in all three patients.

Polymerase chain reaction (PCR) techniques to identify pathogenic bacteria by their genotype has the advantage of identification of bacteria in shorter time, in hours as compared to several days when standard culture techniques are used.^2^ Unfortunately, even with PCR techniques there is high “culture negative” rate. This underlines the fact that detection of causative pathogen in septic arthritis remains challenging.^2^ In all three patients the blood cultures were sterile. However, in Case 1, synovium grew Staphylococcus aureus. PCR testing for micro-organism other than TB is not available at our institution.

The WBC count of more than 50,000 cells/ml in the aspirated joint fluid is considered diagnostic but its value is diminished when the joint aspirate is blood stained,^2^ as was the case in our patients.

ESR and C-RP are common tests done to measure the body's response to the acute inflammatory conditions.^4^ Both tests are non-specific. ESR and C-RP were raised in all 3 patients. However, these tests are more useful when patients present within 24 hours after the onset of symptoms. Their diagnostic value is limited when patients present weeks or months later, as was the case with all three patients. In that situation, these tests are more helpful to monitor the control of the infection following treatment.

Another helpful factor towards the diagnosis of pyogenic infection of the joint is the rapid destruction of bone seen on serial radiographs.^5,6^ In all three patients, radiographs on presentation to the clinic showed marked bony destruction which was not seen on the previous radiographs taken few weeks earlier. However, after the infection is controlled there is considerable reformation of the bone as seen on serial radiographs. Perhaps initial exaggerated appearance of bone destruction could be due to hyperaemia caused by infection.^5,6^

Presence of numerous neutrophil polymorphs on histological examination of synovial tissue is considered important for diagnosis of pyogenic infection and presence of granuloma is suggestive of mycobacterial infection.^2,7^ In all three patients' histopathology was suggestive of pyogenic infection. However, histopathology does not identify nor provide antibiotic sensitivity of the causative micro-organism. Therefore, all the parameters such as clinical features, blood and joint fluid investigations, changes on serial radiographs and histopathology should be correlated to arrive at the clinical diagnosis upon which the treatment is based.^2,7^

Perhaps, it is better to avoid the risk of infection by following strict aseptic precautions when any joint is aspirated or injected.^3,8^ The procedure, preferably should be performed in a clean room and not in the common dressing or treatment rooms where infected cases may have been previously treated.^3,8^ Patients should be frequently followed up after knee injections to enable early recognition of infection to avoid possible progression to osteomyelitis and joint destruction. Physicians should be aware of this uncommon presentation of knee joint infection, in adults, with lack of flagrant signs and symptoms.

## Consent:

**JNMA Case Report Consent Form** was signed by the patient and the original article is attached with the patient's chart.

## Conflict of Interest

**None.**
